# Shielding evaluation and acceptance testing of a prefabricated, modular, temporary radiation therapy treatment facility

**DOI:** 10.1120/jacmp.v5i4.2025

**Published:** 2004-11-24

**Authors:** Gary A. Ezzell

**Affiliations:** ^1^ Department of Radiation Oncology Mayo Clinic Scottsdale 13400 E. Shea Boulevard Scottsdale Arizona 85259 U.S.A.

**Keywords:** radiation shielding, modular structure, temporary vault

## Abstract

We have recently commissioned a temporary radiation therapy facility that is novel in two aspects: it was constructed using modular components, and the LINAC was installed in one of the modular sections before it was lifted into position. Additional steel and granular fill was added to the modular sections on‐site during construction. The building will be disassembled and removed when no longer needed. This paper describes the radiation shielding specifications and survey of the facility, as well as the ramifications for acceptance testing occasioned by the novel installation procedure. The LINAC is a Varian 21EX operating at 6 MV and 18 MV. The radiation levels outside the vault satisfied the design criteria, and no anomalous leakage was detected along the joints of the modular structure. At 18 MV and 600 monitor units (MU) per minute, the radiation level outside the primary barrier walls was 8.5μSv/h of photons; there were no detectable neutrons. Outside the direct‐shielded door, the levels were 0.4μSv/h of photons and 3.0μSv/h of neutrons. The isocentricity of the accelerator met the acceptance criteria and was not affected by its preinstallation into an integrated baseframe and subsequent transport to the building site.

PACS numbers: 87.52.Df, 87.52.Ga

## I. INTRODUCTION

Our department recently began clinical radiation therapy operations in a new facility built with a construction technique that is novel in two important aspects. First, the treatment vault consists of prefabricated modular sections. Second, the LINAC was installed in one of the modules before it was lifted into place. The finished structure is a freestanding building that is designed to be temporary. The accelerator will eventually be relocated to a permanent building, and the modules will then be disassembled. The purpose of this paper is to describe the radiation shielding specifications and survey of the facility, as well as the ramifications for acceptance testing occasioned by the novel installation procedure.

Our department had a pressing need to provide radiation treatment services at a satellite facility. A permanent, multistory building will eventually house the radiation oncology department, but we wished to begin treatments much sooner. Therefore, we began investigating methods of creating a temporary structure. The outcome is the first clinical installation of a new type of modular structure from a vendor of temporary radiation oncology vaults (Rad‐Technology, Pembroke Pines, FL). The modular design offered the possibility of completing construction more rapidly than if traditional methods were used. It also had the advantage of being designed to be disassembled and removed when no longer needed.

The project developed as a collaboration between our institution, the vendor for the building, and the vendor for the LINAC (Varian, Palo Alto, CA). We played no role in the structural design, which is proprietary to the vendor, but we did specify the radiation shielding requirements that the structure would have to meet. The two vendors worked together on how to install the accelerator gantry and stand in one of the modules before it was moved onto our campus. This required incorporating the baseframe into the steel structure of the module. As the customer, we played no role in that design but negotiated acceptance testing agreements with the two vendors that established accountability for the building and accelerator as a combined unit.

## II. METHODS

**Figure 1:**
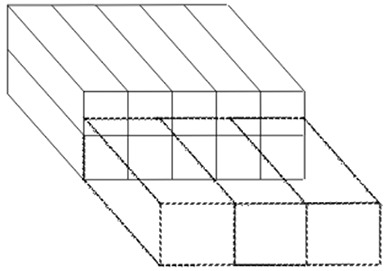
Schematic of modular layout. Solid lines represent the 10 modules that comprise the vault. Dashed lines represent the modules that provide the ancillary work areas.

The treatment vault is composed of two layers of five modules, each 2.4 m (8 ft.) wide, 2.4 m (8 ft.) tall, and 9.8 m (32 ft.) long. The outer dimensions of the vault are therefore 12.2 m (40 ft.) wide, 4.9 m (16 ft.) tall, and 9.8 m (32 ft.) long. The central three modules of the first layer provide the inner usable space, and the outer modules and upper layer incorporate the bulk of the shielding. The treatment vault is mated to three additional modular structures that provide space for the control room; reception, waiting, and dressing areas; offices and examination rooms; and treatment planning. Figure 1 shows a schematic of the arrangement of the modules.

**Figure 2:**
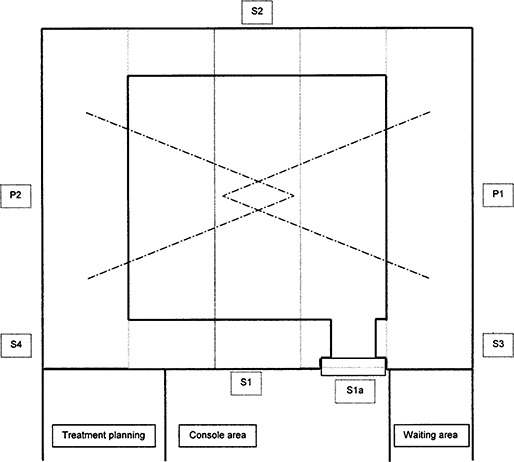
Plan view of structure. Solid lines are finished walls; light gray dashed lines represent boundaries of modular sections; dash‐dot lines represent beam limits. The area designations correspond to Table 1.

The LINAC is a Varian 21EX operating at 6 MV and 18 MV. The vault design (Fig. 2) incorporates a direct shielding door. The treatment vault is a freestanding building that stands on grade and is surrounded by unrestricted, landscaped grounds. The vendor can adjust the proportions of steel plates and proprietary granular fill in the modular walls to control the radiation transmission through the various barriers. Neutron protection is afforded by the granular fill, wood, and borated polyethylene in the walls. The details of the steel and polyethylene placement are proprietary, but the vendor has indicated that, in general, the thickness of steel in the primary beam area is 31.8 cm (12.5 in.), and in the secondary beam area the amount is 21.6 cm (8.5 in.). The thickness of polyethylene varies but is approximately 10.2 cm (4 in.) toward the console area. The door incorporates 15.2 cm (6 in.) of lead, 22.9 cm (9 in.) of borated polyethylene, 2 cm (0.8 in.) of steel, and 1.9 cm (0.75 in.) of plywood.

The minimum radiation shielding was specified using the standard methodology of NCRP^(^ ^1^ ^,^ ^2^ ^)^ in order to meet the regulatory requirements of Arizona. The maximum barrier transmission was calculated for all individual primary and secondary barriers, taking into account the state requirements for restricted and unrestricted areas and the relevant hourly and annual limits. For unrestricted areas, those limits are 1000μSv/yr(0.1rem/yr) assuming average occupancy and 20 μSv (2 mrem) in any one hour assuming continuous occupancy. A conservative estimate of workload was applied: 60 000 MU/week entirely delivered by 18 MV. For the secondary barriers, the workload was increased by a factor of 2.5 to account for intensity‐modulated radiotherapy (IMRT) delivery.^(^ ^3^ ^)^ This is a conservative workload selection since IMRT is actually delivered at 6 MV.

The roof required special consideration. Previous demonstration installations of the modular vault in Florida and Canada had provided only minimum shielding for the roof. A similar design could have been used for this freestanding structure, but positive access control to the roof would have been necessary. We preferred to shield the roof to a level that would make it a “Radiation Area,” meaning that an individual continuously occupying the area might receive between 50 μSv and 1000 μSv (5–100 mrem) in one hour. Taking as a worst case that the beam might be directed upward during maintenance for one‐quarter of an hour, this design limit corresponded to a permissible continuous rate of up to 4mSv/h(400mrem/h). The primary beam area on the roof (plus a margin) was marked and signed as a Radiation Area. Given the difficulty of accessing the roof and the expectation that no access would be necessary for the life of this temporary structure, this solution was deemed satisfactory and was accepted by the state regulators. The area outside the barrier, called S5 in Table 1, was held to a limit of 20 μSv in any one hour with the same assumptions.

**Table 1:** Design summary for the radiation barriers, assuming full use at 18 MV and with the workloads described in the text. The “use factor” refers to the fraction of the time the beam is directed at the barrier. For the calculation based on limits per hour, the use factor is taken to be one, and the “duty factor” refers to the fraction of one hour that the beam may be on. The required meters of concrete (or equivalent) is given for the limiting case, using tenth‐value layers as described in the text.

Barrier ID	Location	Barrier type	Area type	Workload period	Limit (μSv)	Use factor	Occup. factor	Duty factor	Trans. factor	Meters o concrete
P1, P2	side walls	1°	uncontrolled	per week	1000	1/4	1/4		2.69E‐05	
				per hour (TBI)	20	1	1	1/5	1.40E‐05	
				per hour (continuous)	20	1	1	1	2.80E‐06	2.47
P1a, P2a	above 3.2 m	1°	uncontrolled	per week	1000	1/4	1/16		1.34E‐04	1.76
				per hour	20	1	1	0.025	1.12E‐04	
P3	roof	1°	controlled	per hour	1000	1	1	1/4	2.57E‐04	1.60
S1	front wall	2°	controlled	per week	100	1	1		1.37E‐03	1.13
S1a	door	2°	controlled	per week	100	1	1		1.37E‐03	1.13
S2	back wall	2°	uncontrolled	per week	20	1	1/4		1.13E‐03	1.16
S3,4	side walls	2°	uncontrolled	per week	20	1	1/4		2.23E‐03	1.09
S5	roof	2°	uncontrolled	per week	20	1	1/16		3.48E‐03	
				per hour	20	1	1	1/4	2.29E‐03	1.04

TBI = total body irradiation.

For the primary barriers forming the side walls, P1 and P2, average conditions were considered for the weekly limit. For the hourly limit, the special situations of total body irradiation and beam scanning were also considered.

Another area of special interest, labeled P1a, P2a in Table 1, is the region in the primary beam along the walls at a height of more than 3.2 m (10 ft.) above the ground. This is another area that could be reached only by ladder and did not justify the expense of full shielding. The shielding calculation for the hourly limit assumed a worst case of six patients treated during an hour with half of their fields oriented obliquely upward, delivering 900 MU in that direction. Expressed as a fraction of continuous irradiation at 600 MU/min, this corresponds to a duty factor of 0.025.

Table 1 identifies the major barriers and elements of the shielding calculations. The required barrier thickness expressed as standard density concrete is also shown for the limiting transmission factor. This was determined using tenth‐value layers of 44.5 cm (17.5 in.) for the primary 18‐MV beam and 39.4 cm (15.5 in.) for the 18‐MV leakage at 90° as per NCRP 51.

The barrier transmission factors in Table 1 determined the maximum acceptable ambient radiation levels that could be measured during the radiation survey with the 18‐MV beam running continuously at 600 MU/min. Those values are listed in Table 2 as “design limits.”

**Table 2:** Radiation barriers, design limits, and measured values for dose equivalent rates with the accelerator producing 18 MV at 600 MU/min. These rates are for the worst‐case irradiation condition (e.g., for a primary barrier, the beam is directed at the barrier). The design limit is the predicted total dose equivalent rate for the barrier using the limiting transmission factor from Table 1.

Barrier ID	Location	Design limit μSv/h	Measured photons μSv/h	Measured neutrons μSv/h	Total μSv/h
P1, P2	side walls	20	8.5	<0.5	<9.0
P1a, P2a	above 3.2 m	800	300	<0.5	300
P3	roof	4000	2200	40	2240
S1	front wall	24	0.1	2.0	2.1
S1a	door	24	0.4	3.0	3.4
S2	back wall	19	0.3	<0.5	<0.8
S3, 4	side walls	19	1.7	<0.5	<2.2
S5	roof	80	25	<0.5	25

The vendor supplied drawings for review that specified the barrier thicknesses in terms of equivalent thicknesses of standard density concrete at 18 MV. The vendor chose to exceed our design requirements for this leased, reusable structure. The vendor also supplied documentation of radiation surveys of the two demonstration facilities that had been set up in order to achieve licensing in Florida and Canada. These documents were used for our preconstruction design analysis and state submittal.

Our contract with the building vendor specified certain acceptance conditions. Among them were two provisions related to physics. First, the measured ambient radiation levels needed to meet the shielding criteria. Second, the LINAC had to pass the components of the accelerator acceptance test procedure related to isocentricity. The purchase agreement with the accelerator vendor specified its standard acceptance test document. This combination protects us as the customer should the two vendors not collaborate successfully in producing an installation that worked and was mechanically stable, and it isolated each vendor against problems unrelated to its portion of the project.

After construction of the treatment vault and control area, the accelerator vendor completed the installation of the treatment unit. A preliminary radiation survey was first done to assure that the installation could be completed safely, and a detailed radiation survey was performed afterward. The survey utilized an ionization chamber (Victoreen 450P) and a neutron remmeter (Ludlum 05‐711). A counter equipped with a scintillation probe was used to search for possible localized radiation leaks. Special attention was paid to areas where the modules were joined together.

## III. RESULTS

**Figure 3:**
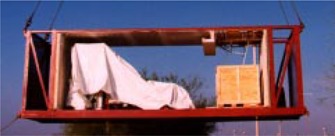
The central module with the LINAC being lifted into position

Figure 3 shows module 3 being lifted into place with the accelerator already fixed to the integrated baseframe. Over the course of several days, the modules were placed and bolted together and the additional steel and granular fill added to the structure. When the internal finishing was sufficiently complete, the accelerator vendor completed the installation of the treatment unit.

The acceptance test for the LINAC was completed without difficulty, with the measured mechanical and radiation isocenter having a radius ≤0.5mm. No degradation of the isocentricity has been noted in the initial 6 months of use.

The full radiation safety survey was completed satisfactorily following a preliminary calibration of the treatment beam. The survey was performed for both photon energies and the full range of gantry angles and while scaffolding was in place around the exterior for the attachment of the external finishes. Table 2 shows the maximum measured dose equivalent rates for selected points outside the various barriers, obtained with the accelerator producing 18 MV at 600 MU/min. No areas of anomalous leakage were detected using the scintillation counter, indicating that the joints were properly overlapped and the fill had no voids.

## IV. DISCUSSION

Some elements of the shielding design were quite conservative, such as assuming that the entire workload would use 18 MV delivered with IMRT. Some of these decisions were predicated on the surveys of the previous demonstration installations of the modular vault, which was designed to meet international standards that are, in some cases, more restrictive than are common in the United States. Thus, we were able to specify stringent criteria with confidence that the vendor had already met them. Also, the expense of fabricating the leased structure did not affect the cost to our institution, removing a consideration that normally influences the design of permanent structures. The preexisting roof design, on the other hand, was not suitable for our needs, and so required a more realistic balancing of the cost and risk. The chosen solution was reasonable given the inaccessibility of the roof and was acceptable to the state regulators.

One reason for employing the modular construction method was to reduce construction time, and some savings were realized. Our institution's senior project manager has estimated that using the modular structures saved an estimated 4 to 5 months of construction time compared to conventional building methods. The assembly of the modules on the foundation pad and the addition of the shielding materials were completed in approximately one week. Interior finishing and accelerator installation followed, with the acceptance test occurring eight weeks after beginning to place the modules. The only part of the LINAC installation that took place prior to construction was the mounting of the gantry and stand on the integrated baseframe. The counterweight was placed in the central module before it was lifted into place and was attached to the gantry afterward. Installation of the treatment table, modulator, and control systems occurred in the normal fashion after the construction was completed. Patient treatments began nine weeks after acceptance testing, following beam commissioning, additional interior and exterior finishing, and other clinical preparations.

## V. CONCLUSION

The radiation safety and mechanical stability requirements of the facility were satisfied using this novel construction method, which reduced the overall construction time of the project.
